# Serum 11-ketotestosterone is associated with overall disease-control status but adds limited information beyond androstenedione in children with 21-hydroxylase deficiency

**DOI:** 10.3389/fendo.2026.1913565

**Published:** 2026-09-08

**Authors:** Tian Lan, Wen Ruan, Kaibi Chen, Meng Yang, Xiaohong Chen, Hui Yao

**Affiliations:** 1 Department of Genetics, Metabolism and Endocrinology, Wuhan Children’s Hospital, Tongji Medical College, Huazhong University of Science and Technology, Wuhan, China; 2 Department of Central Laboratory, Wuhan Children’s Hospital, Tongji Medical College, Huazhong University of Science and Technology, Wuhan, China

**Keywords:** 11-Ketotestosterone, 21-hydroxylase deficiency, congenital adrenal hyperplasia, exploratory association, liquid chromatography-tandem mass spectrometry

## Abstract

**Introduction:**

Monitoring children with 21-hydroxylase deficiency (21-OHD) requires suppression of adrenal androgen excess while avoiding glucocorticoid overtreatment. However, the clinical value of serum 11-oxygenated androgens beyond established biomarkers remains uncertain. We evaluated whether serum 11-ketotestosterone (11KT) is associated with overall disease-control status and whether it provides incremental information beyond androstenedione (A4).

**Methods:**

We conducted a retrospective, single-center study of 67 children with classic 21-OHD and 67 healthy controls. Steroids were measured by liquid chromatography–tandem mass spectrometry in fasting serum samples collected at 08:00 before medication. Two pediatric endocrinologists, masked to all steroid results, independently adjudicated overall disease-control status using clinical information from the preceding 12 months. Associations of 11KT with disease status and A4 were examined after adjustment for relevant covariates. Discrimination was assessed using receiver operating characteristic analysis, and the incremental value of 11KT beyond age, sex, Tanner group, and A4 was evaluated using bootstrap-corrected ridge regression models.

**Results:**

Serum 11KT was higher in children with 21-OHD than in healthy controls (adjusted geometric mean ratio, 1.81; 95% CI, 1.24–2.64; P = 0.002) and in suboptimally controlled than in well-controlled children (6.17; 95% CI, 3.18–11.97; P < 0.001). Within the 21-OHD group, 11KT was strongly correlated with A4 after adjustment for age, sex, and Tanner group (partial r = 0.843). The single-marker area under the receiver operating characteristic curve (AUC) was 0.875 for 11KT and 0.906 for A4. Adding 11KT to a ridge model containing age, sex, Tanner group, and A4 changed the bootstrap-corrected AUC from 0.912 to 0.903 and the corrected calibration slope from 0.835 to 0.695. Lower-limit sensitivity analyses yielded the same qualitative findings.

**Discussion:**

Serum 11KT was associated with physician-adjudicated overall disease-control status in children with classic 21-OHD, but a single morning measurement provided no stable incremental information beyond A4 in this cohort. These findings do not support replacing A4 with 11KT or using 11KT as a standalone treatment target. Its potential clinical value may instead lie in contextual interpretation, particularly when conventional biomarkers and clinical findings are discordant.

## 1 Introduction

Long-term management of pediatric 21-hydroxylase deficiency (21-OHD), the most common form of congenital adrenal hyperplasia (CAH), requires a practical balance: adrenal androgen excess should be suppressed, but glucocorticoid overtreatment must be avoided. Insufficient control can accelerate skeletal maturation, trigger central precocious puberty or progressive virilization, and compromise adult height. Excessive glucocorticoid exposure, in contrast, may slow linear growth and increase metabolic risk. Treatment monitoring in children therefore depends on a combined reading of biochemical markers, growth, pubertal development, bone-age progression, medication timing, and clinical safety (1, 2).

Conventional biomarkers only partly meet this need. Serum 17-hydroxyprogesterone (17-OHP) is affected by circadian rhythm, stress, and the timing of hydrocortisone dosing, so a single measurement may not reflect overall androgen exposure. Androstenedione (A4) and testosterone (T) are clinically useful, but their interpretation becomes more difficult during puberty because gonadal steroid production contributes to circulating concentrations. Disagreement among biomarkers, or between biomarkers and growth or virilization, remains a familiar problem in routine care (3–5).

Adrenal-derived 11-oxygenated androgens have emerged as biologically relevant markers in 21-OHD. In classic 21-OHD, these C19 steroids form a major circulating androgen pool and have been associated with adrenal volume and testicular adrenal rest tumors (6, 7). Treated children with androgen excess also have higher 11-oxygenated androgen concentrations (8). Pediatric reference intervals and recent monitoring studies further indicate that age, sex, and pubertal status must be considered when interpreting these steroids (4, 9–12).

However, the incremental value of serum 11-ketotestosterone (11KT) beyond established biomarkers, particularly A4 and 17-OHP, remains unclear in children with 21-OHD. We therefore examined the cross-sectional association between a single fasting morning 11KT concentration and physician-adjudicated overall disease-control status assessed using clinical information collected over the preceding 12 months. We also tested whether 11KT added information to a model containing age, sex, Tanner group, and A4.

## 2 Materials and methods

### 2.1 Study population

This retrospective, single-center study included children with 21-OHD who were followed at Wuhan Children’s Hospital from February 2021 to February 2022. Children with classic salt-wasting or simple-virilizing phenotypes were eligible; those with non-classic 21-OHD were excluded. The comparison group comprised healthy children undergoing routine physical examinations. Age, sex, and Tanner stage were recorded, and Tanner stage was categorized as 1 or ≥2 for comparative and stratified analyses.

The diagnosis of 21-OHD was based on clinical manifestations, biochemical assessment, and genetic testing according to the 2018 Endocrine Society guideline (1). The study was approved by the Ethics Committee of Wuhan Children’s Hospital (2022R081-E01), and written informed consent was obtained from the legal guardians of the enrolled children. All methods were performed in accordance with relevant guidelines and regulations.

### 2.2 Follow-up and steroid measurement

The enrolled children were followed every 3 months. At each visit, height, weight, secondary sexual characteristics, signs of glucocorticoid excess, adrenal crisis history, and medication were recorded. All patients received hydrocortisone at a total daily dose of 10–15 mg/m²/day, administered orally in three divided doses. Patients with the salt-wasting phenotype and some patients with the simple-virilizing phenotype and elevated renin levels also received fludrocortisone (0.05–0.2 mg/day). Venous blood (2 mL) was collected at 08:00 after overnight fasting and before morning medication, placed in an inert separation-gel coagulation tube, and centrifuged within 10 min of collection. Serum was held at 4 °C for no longer than 4 h, transferred to −80 °C, and subjected to one freeze–thaw cycle before liquid chromatography–tandem mass spectrometry (LC–MS/MS) analysis. Bone-age radiography was performed every 6 months, and male patients underwent testicular ultrasonography to screen for testicular adrenal rest tumors.

Serum specimens collected during follow-up were stored at −80 °C and analyzed for 11β-hydroxyandrostenedione (11OHA4), 11KT, and 11-ketodihydrotestosterone (11KDHT) in March 2022. The results were unavailable to treating clinicians and did not influence treatment or endpoint classification. To limit incorporation and information bias, adjudicators used only serial clinical records, growth measurements, bone-age reports, complications, treatment exposure, and routine assessments from the 12 months preceding and including the index visit. All steroid results remained masked until the endpoint database was locked.

Serum concentrations of 17-OHP, A4, T, progesterone (PROG), 11OHA4, 11KT, and 11KDHT were measured using ultra-high-performance liquid chromatography–tandem mass spectrometry (UHPLC–MS/MS) with a Shimadzu LCMS-8050CL system (Shimadzu Corporation, Kyoto, Japan).

Chromatographic separation was performed using a Shim-pack GIST C18 column (2 μm, 100 mm × 2.1 mm). The mobile phases consisted of 0.1% formic acid in water (phase A) and 0.1% formic acid in methanol (phase B). Gradient elution was performed at a flow rate of 0.2–0.4 mL/min with the column temperature maintained at 40 °C. Detection was conducted in positive electrospray ionization (ESI) mode using multiple reaction monitoring (MRM).

Steroid quantification was performed using a stable isotope dilution method. Calibration standards were prepared using serum samples spiked with mixed steroid standards at different concentrations, and stable isotope-labeled internal standards were added to all calibration samples and clinical specimens. Serum samples underwent methyl tert-butyl ether extraction, followed by nitrogen evaporation, methanolic reconstitution, centrifugation, and injection into the UHPLC–MS/MS system.

Calibration curves were generated using the analyte-to-internal standard concentration ratio plotted against the corresponding peak-area ratio using LabSolutions software (Shimadzu Corporation, Kyoto, Japan). Weighted linear regression was applied, with acceptable linearity defined as R² > 0.99.

Analytical performance was assessed using precision and recovery experiments. Intra-assay precision was evaluated using low-, medium-, and high-level quality-control samples measured in replicate, and inter-assay precision was assessed over 15 days. The intra- and inter-assay coefficients of variation were < 15%. Recovery was evaluated using spiked pooled serum samples at three concentration levels, with mean recovery ranging from 85.33% to 118.81%.

Routine quality control was performed using stable isotope dilution-based monitoring. Acceptance criteria included internal-standard peak-area deviation within ± 15%, retention-time deviation within ± 0.3 min, absence of analyte peaks in blank samples, substitute-matrix accuracy within 80%–120%, and quality-control sample deviation within ± 15%.

### 2.3 Clinical assessment

Bone age (BA) was assessed by trained pediatric endocrinologists using the Greulich–Pyle atlas (13). Height standard deviation scores (SDS) for chronological age were calculated using Chinese growth standards for children and adolescents aged 0–18 years (14). Annualized height velocity was calculated from two standing-height measurements separated by approximately 12 months as the height difference divided by the interval in years. Annualized bone-age progression was calculated from radiographs obtained 6–12 months apart as the change in BA divided by the change in chronological age (ΔBA/ΔCA).

### 2.4 Definition of overall disease-control status

Children with 21-OHD were classified as well controlled or suboptimally controlled using a prespecified composite clinical definition. The endpoint was physician-adjudicated overall disease-control status, retrospectively assessed using clinical information collected over the preceding 12 months. Well-controlled status required fulfillment of all five criteria during the observation period: (1) no deepening of skin pigmentation and no newly emerged or progressive virilization, including acne, hirsutism, clitoral enlargement, or penile enlargement; (2) no clinical signs of glucocorticoid excess; (3) no adrenal crisis, central precocious puberty, or testicular adrenal rest tumor; (4) normal linear growth (annual Δheight SDS for chronological age −0.3 to +0.3), and (5) appropriate bone-age progression (ΔBA/ΔCA < 1.25) (1, 2, 5, 9); the numerical growth and bone-age thresholds were prespecified for this study. Patients who failed to meet any one of these criteria were classified as suboptimally controlled.

Clinical control status was independently adjudicated by two pediatric endocrinologists blinded to steroid measurements using standardized clinical summaries. Discordant assessments were reviewed by a third endocrinologist for final classification, and the endpoint database was locked before steroid results were unblinded.

### 2.5 Statistical analysis

Statistical analyses, tables, and figures were generated in Python 3.12.13 using NumPy, pandas, SciPy, statsmodels, scikit-learn, Matplotlib, and seaborn. Continuous and categorical variables were compared using Mann–Whitney U and Fisher’s exact tests, respectively. Models comparing patients with controls were adjusted for age, sex, and Tanner group; models comparing overall disease-control groups were additionally adjusted for phenotype. Adjusted geometric mean ratios were obtained from ordinary least-squares models of log2-transformed concentrations with heteroskedasticity-consistent type 3 (HC3) standard errors. Spearman’s and partial correlations were reported with percentile bootstrap 95% confidence intervals; partial correlations were adjusted for age, sex, and Tanner group.

The prespecified primary incremental analysis compared a ridge logistic model containing age, sex, Tanner group, and log2(A4) with the same model plus log2(11KT). Ridge regression used a fixed penalty of λ = 1 and an unpenalized intercept. Discrimination was quantified using the area under the receiver operating characteristic curve (AUC), and paired AUCs were compared using DeLong tests. Internal validation used 1,000 bootstrap resamples with complete model refitting and a fixed random seed. The primary analysis replaced 11KT values below the lower limit of quantification (LLOQ) with LLOQ/√2; LLOQ/2 substitution and exclusion were sensitivity analyses. Subgroup analyses were exploratory because of limited stratum-specific sample sizes.

## 3 Results

### 3.1 Baseline characteristics

The analysis included 67 children with 21-OHD and 67 healthy controls. Age (7.83 [4.71, 10.25] versus 9.25 [6.42, 10.25] years; P = 0.260), sex distribution (male/female participants, 40/27 versus 35/32; P = 0.486), and Tanner group (stage 1/≥2, 35/32 versus 31/36; P = 0.604) were similar between groups (**Table 1**).

**Table 1:** Baseline characteristics of children with 21-OHD and healthy controls.

Characteristic	Children with 21-OHD (n = 67)	Healthy controls (n = 67)	Test	P-value
Age, years	7.83 [4.71, 10.25]	9.25 [6.42, 10.25]	Mann–Whitney U	0.260
Sex, male/female participants	40/27	35/32	Chi-square with continuity correction	0.486
Tanner group, Tanner 1/Tanner ≥2	35/32	31/36	Chi-square with continuity correction	0.604

### 3.2 Steroid concentrations in 21-OHD and healthy controls

**Figure 1:**
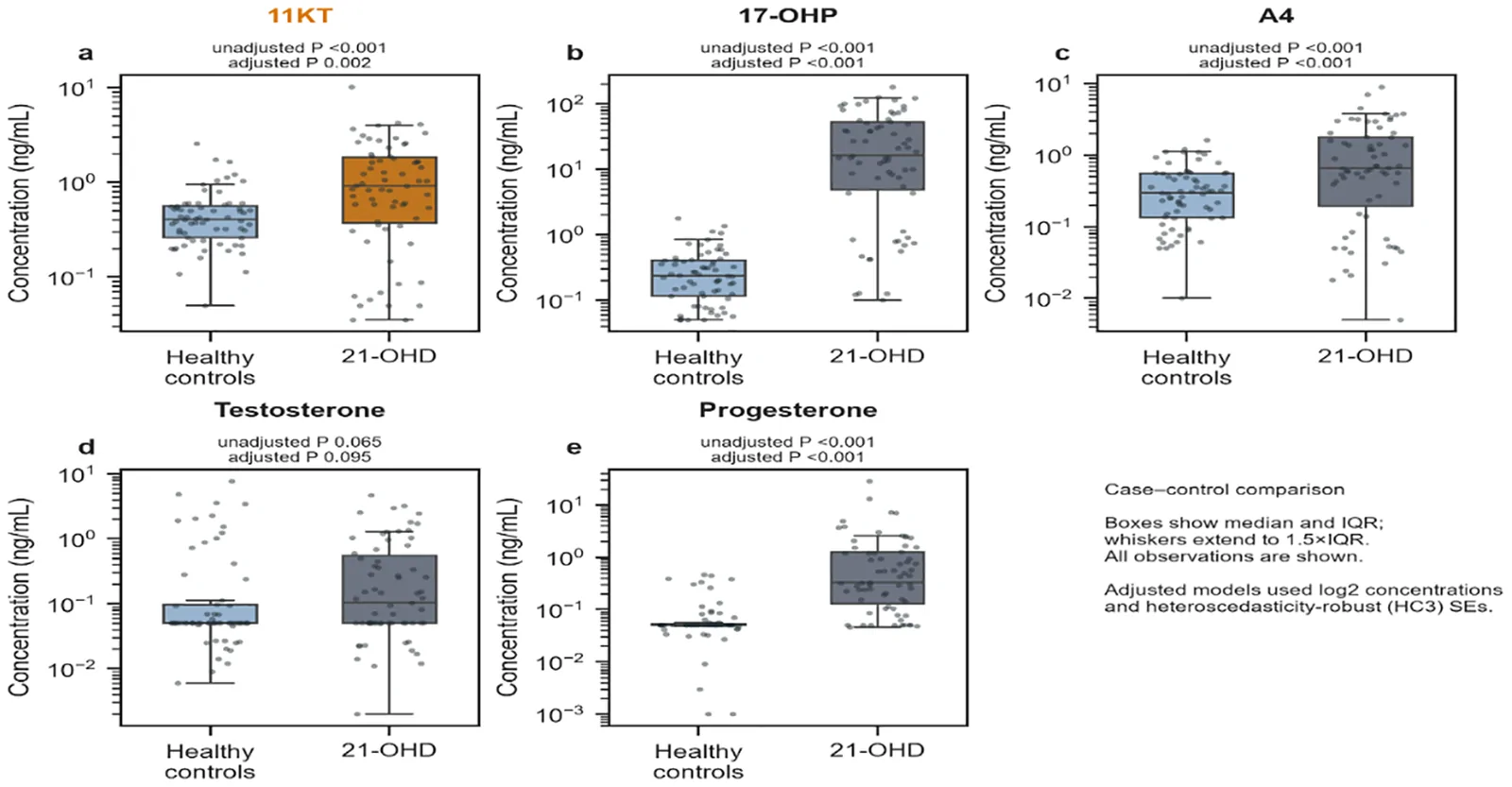
Validated steroid concentrations in children with 21-OHD and healthy controls. Panels **(a–e)** show 11KT, 17-OHP, A4, testosterone, and progesterone, respectively, on log10 scales; all observations are overlaid. Unadjusted P values are shown within panels. Adjusted comparisons are geometric mean ratios from log2-scale models with age, sex, Tanner group, and HC3-robust standard errors.

Serum 11KT was higher in children with 21-OHD than in healthy controls (0.910 [0.372, 1.843] versus 0.406 [0.260, 0.557] ng/mL; P < 0.001; **Figure 1**; **Table 2**). After adjustment for age, sex, and Tanner group, the geometric mean ratio was 1.81 (95% CI, 1.24–2.64; P = 0.002). Adjusted concentrations of 17-OHP, A4, and PROG were also higher in 21-OHD, whereas T did not differ significantly (geometric mean ratio, 1.59; 95% CI, 0.92–2.73; P = 0.095).

**Table 2:** Steroid concentrations in children with 21-OHD and healthy controls.

Variable	21-OHD (n = 67)	Healthy controls (n = 67)	Unadjusted P	Adjusted GMR (95% CI); P
11KT	0.910 [0.372, 1.843]	0.406 [0.260, 0.557]	< 0.001	1.81 (1.24–2.64); 0.002
17-OHP	16.204 [4.858, 51.991]	0.236 [0.115, 0.407]	< 0.001	50.23 (28.95–87.15); < 0.001
A4	0.654 [0.193, 1.772]	0.297 [0.135, 0.552]	< 0.001	2.21 (1.43–3.40); < 0.001
T	0.103 [0.050, 0.547]	0.050 [0.050, 0.097]	0.065	1.59 (0.92–2.73); 0.095
PROG	0.329 [0.129, 1.228]	0.050 [0.050, 0.053]	< 0.001	8.54 (5.32–13.71); < 0.001

### 3.3 Steroid concentrations by overall disease-control status

**Figure 2:**
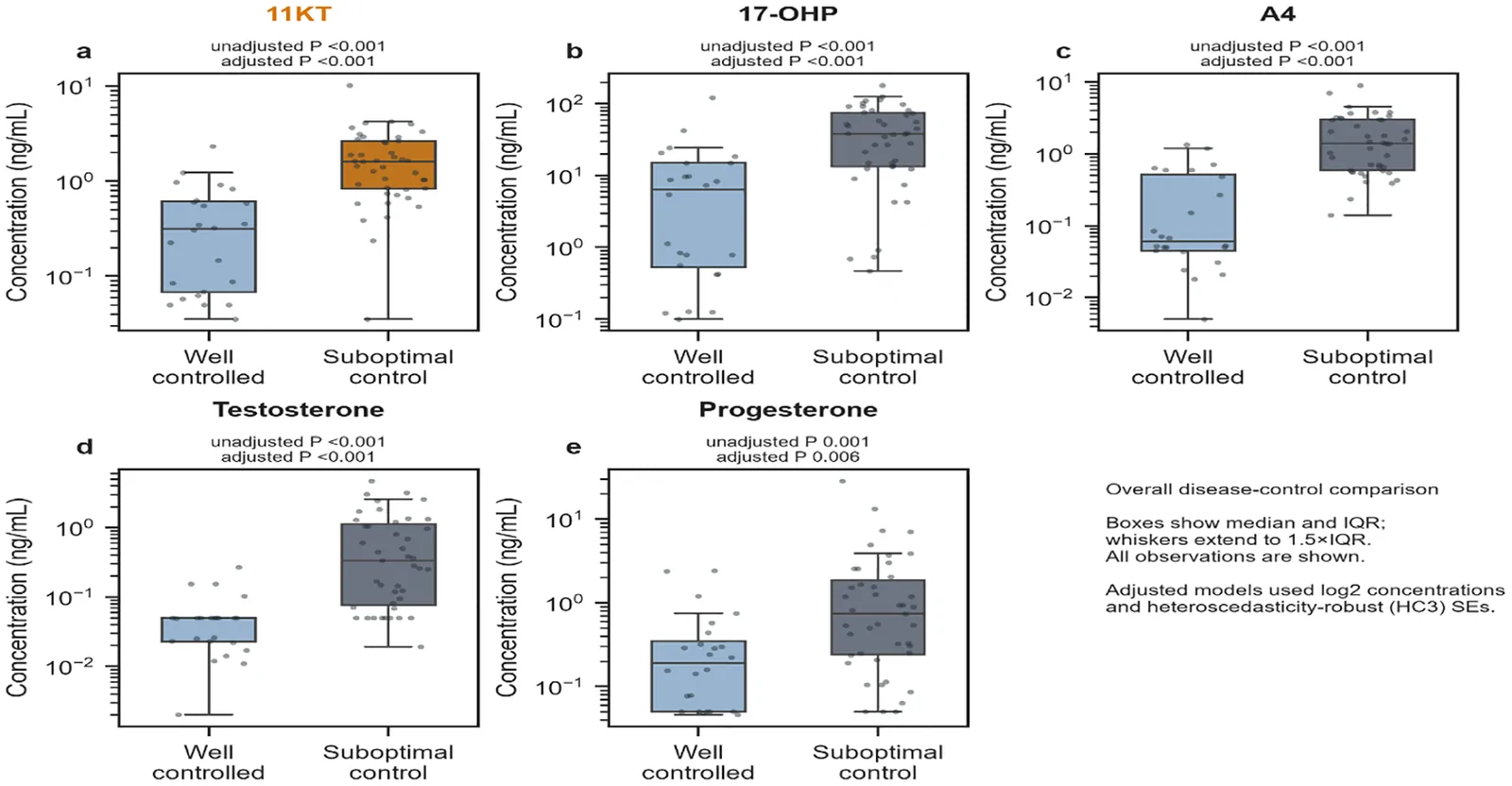
Validated steroid concentrations according to overall disease-control status in children with 21-OHD. Panels **(a–e)** show 11KT, 17-OHP, A4, testosterone, and progesterone, respectively, on log10 scales; all observations are overlaid. Adjusted comparisons account for age, sex, Tanner group, and CAH phenotype.

Of the 67 children with 21-OHD, 24 were well controlled and 43 were suboptimally controlled. Median 11KT was higher in the suboptimally controlled group (1.616 [0.828, 2.636] versus 0.314 [0.068, 0.610] ng/mL; P < 0.001; **Figure 2**; **Table 3**). After adjustment for age, sex, Tanner group, and phenotype, the geometric mean ratio was 6.17 (95% CI, 3.18–11.97; P < 0.001). Concentrations of 17-OHP, A4, T, and PROG were higher in the same direction.

**Table 3:** Steroid concentrations according to overall disease-control status in children with 21-OHD.

Variable	Suboptimally controlled (n = 43)	Well controlled (n = 24)	Unadjusted P	Adjusted GMR (95% CI); P
11KT	1.616 [0.828, 2.636]	0.314 [0.068, 0.610]	< 0.001	6.17 (3.18–11.97); < 0.001
17-OHP	37.974 [13.392, 74.325]	6.372 [0.527, 14.874]	< 0.001	8.82 (2.84–27.38); < 0.001
A4	1.388 [0.589, 2.994]	0.060 [0.044, 0.508]	< 0.001	7.35 (3.49–15.45); < 0.001
T	0.336 [0.076, 1.120]	0.050 [0.023, 0.050]	< 0.001	4.36 (2.39–7.98); < 0.001
PROG	0.740 [0.241, 1.857]	0.190 [0.050, 0.349]	0.001	3.07 (1.38–6.83); 0.006

The cohort comprised 42 children with the salt-wasting phenotype (62.7%) and 25 with the simple-virilizing phenotype (37.3%). Among the 43 suboptimally controlled children, abnormal bone-age progression was the most frequent unmet component (27/43, 62.8%), followed by a major disease-related complication (20/43, 46.5%), abnormal linear growth (13/43, 30.2%), clinical glucocorticoid excess (10/43, 23.3%), and uncontrolled virilization (9/43, 20.9%). Eighteen children met one suboptimal-control component, 14 met two, and 11 met three; mutually exclusive patterns are reported in **Supplementary Table S1**. Phenotype distributions are shown in **Table 4**.

**Table 4:** Baseline characteristics according to overall disease-control status in children with 21-OHD.

Variable	Well controlled (n = 24)	Suboptimally controlled (n = 43)	P-value
Baseline features			
CAH phenotype, n (%)			0.064
Classic salt-wasting (SW)	19/24 (79.2%)	23/43 (53.5%)	—
Classic simple-virilizing (SV)	5/24 (20.8%)	20/43 (46.5%)	—
Age, years	4.92 [3.25, 6.71]	9.42 [5.83, 10.92]	< 0.001
Tanner stage	1 [1, 1.25]	2 [1, 3]	0.003
Sex, male participants, n (%)	14/24 (58.3%)	26/43 (60.5%)	1.000
Δheight/year	6.7 [6.00, 7.67]	6.7 [3.25, 8.85]	0.336

The two initial adjudicators agreed in 64 of 67 cases (95.5%; Cohen’s κ = 0.904). The remaining three cases underwent third-expert adjudication (**Supplementary Table S2**).

### 3.4 Correlation analyses

**Figure 3:**
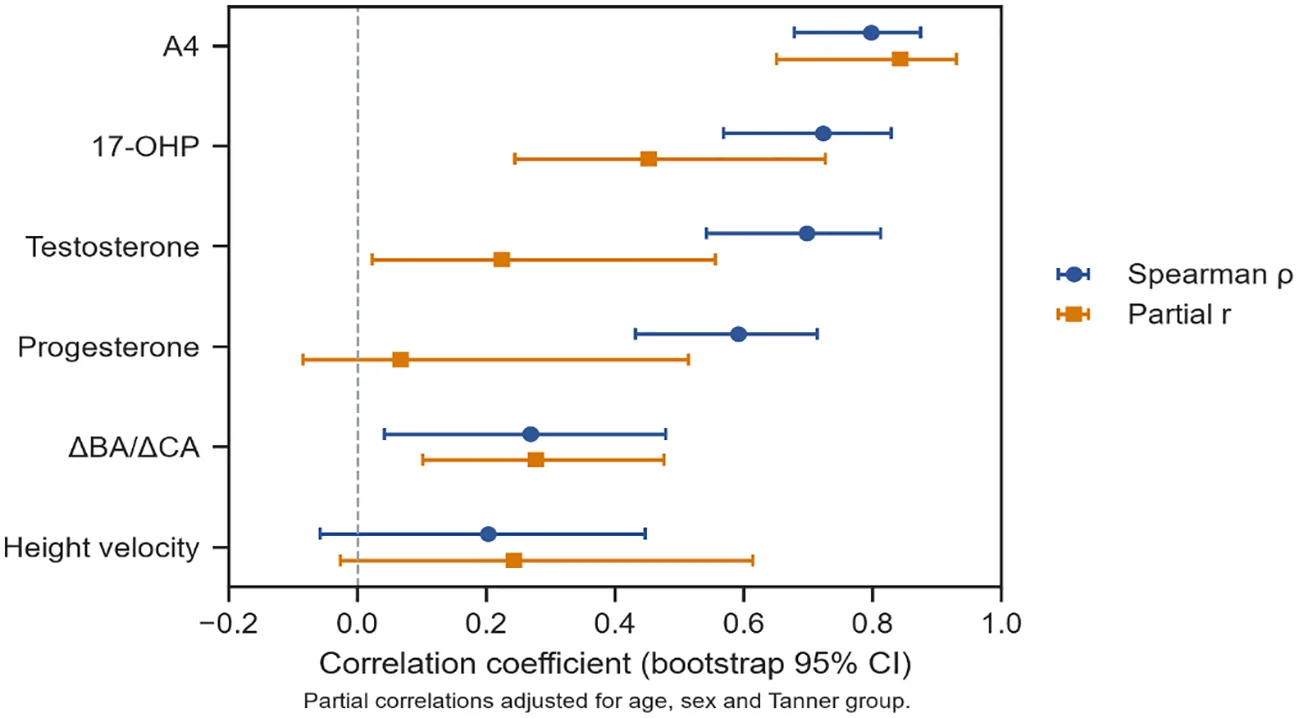
Correlations between 11KT and validated conventional biomarkers or clinical indices. Points show Spearman’s ρ and partial r with bootstrap 95% CIs; partial correlations are adjusted for age, sex, and Tanner group. Both 11OHA4 and 11KDHT are excluded from inferential displays because analyte-specific validation was unavailable.

In the primary LLOQ/√2 analysis, 11KT correlated most strongly with A4 (Spearman’s ρ = 0.798; bootstrap 95% CI, 0.679–0.874) and 17-OHP (ρ = 0.723; 95% CI, 0.568–0.829; **Table 5**; **Figure 3**). After adjustment for age, sex, and Tanner group, the partial correlations were 0.843 (95% CI, 0.651–0.930) for A4 and 0.452 (0.244–0.726) for 17-OHP. The partial correlation with ΔBA/ΔCA progression was 0.277 (0.101–0.476).

**Table 5:** Correlations between 11KT and conventional biomarkers or clinical indices in children with 21-OHD.

Variable	Spearman’s ρ	Spearman P	Partial r	Partial P
A4	0.798 (0.679–0.874)	< 0.001	0.843 (0.651–0.930)	< 0.001
17-OHP	0.723 (0.568–0.829)	< 0.001	0.452 (0.244–0.726)	< 0.001
T	0.697 (0.542–0.812)	<0.001	0.224 (0.023–0.556)	0.075
PROG	0.591 (0.432–0.714)	<0.001	0.067 (−0.084–0.514)	0.600
ΔBA/ΔCA	0.269 (0.042–0.479)	0.028	0.277 (0.101–0.476)	0.026
Height velocity	0.202 (−0.058–0.447)	0.101	0.244 (−0.026–0.614)	0.052

### 3.5 Exploratory discrimination and incremental-value analyses

**Figure 4:**
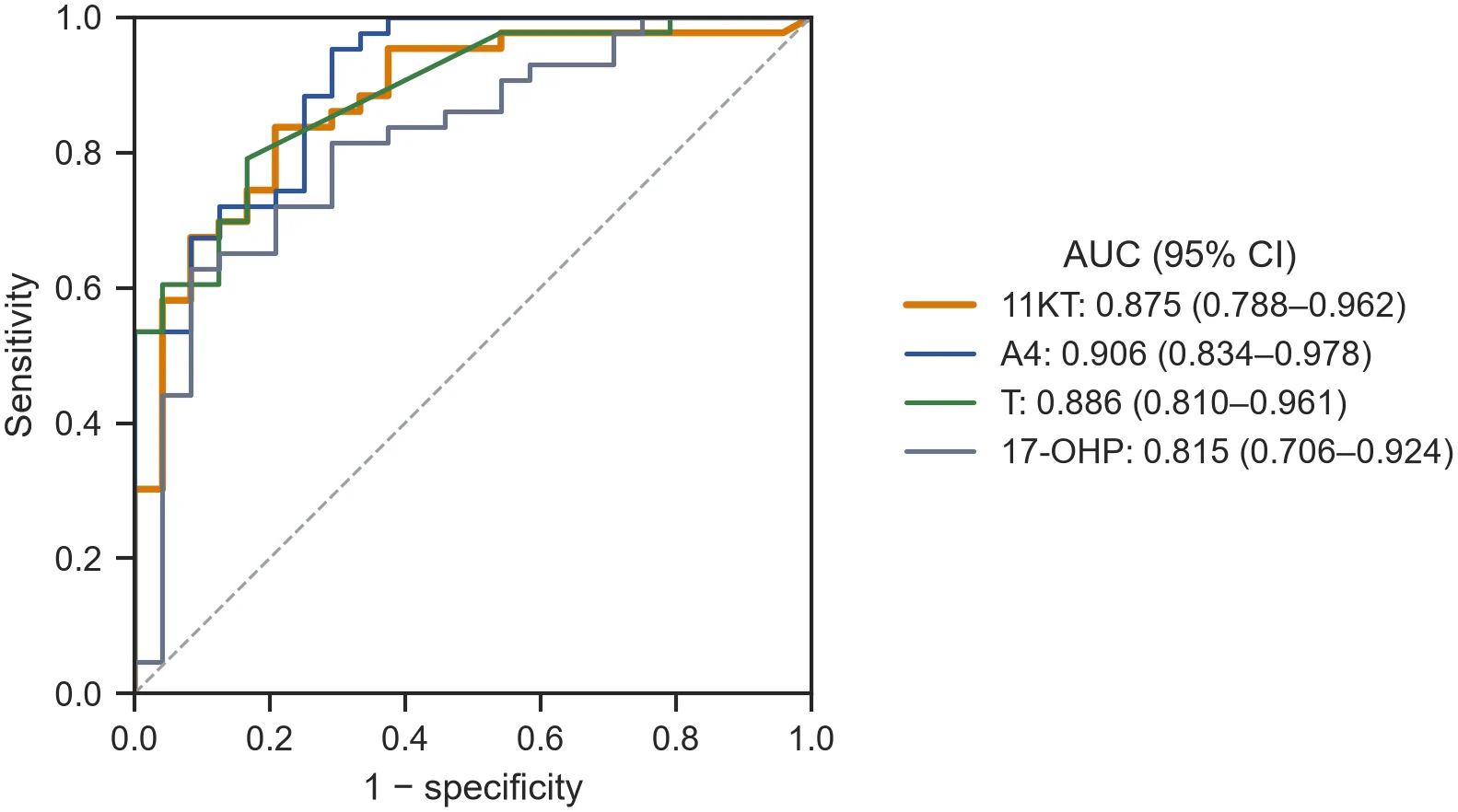
Single-marker receiver operating characteristic (ROC) curves for the cross-sectional overall disease-control endpoint. The 11KT AUC was 0.875 (95% CI, 0.788–0.962); A4, testosterone, and 17-OHP had AUCs of 0.906, 0.886, and 0.815, respectively. Paired DeLong comparisons with 11KT were not statistically significant. The in-sample 0.662 ng/mL Youden’s cutoff is exploratory and is not a transferable clinical threshold.

**Figure 5:**
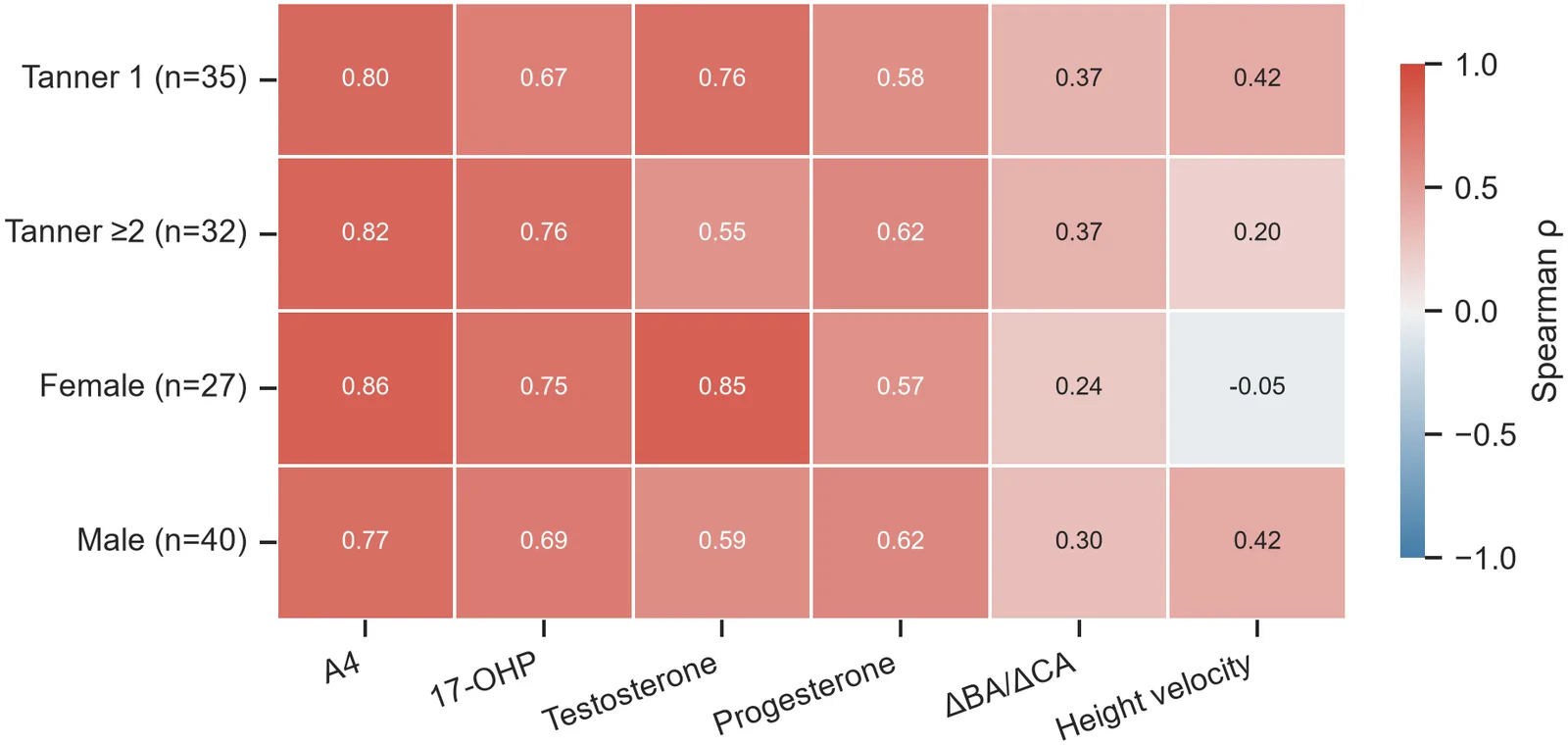
Stratified Spearman’s correlations of 11KT with validated conventional biomarkers and clinical indices by Tanner group and sex. Heatmap annotations show ρ. Subgroup analyses are exploratory because of smaller stratum-specific sample sizes.

**Figure 6:**
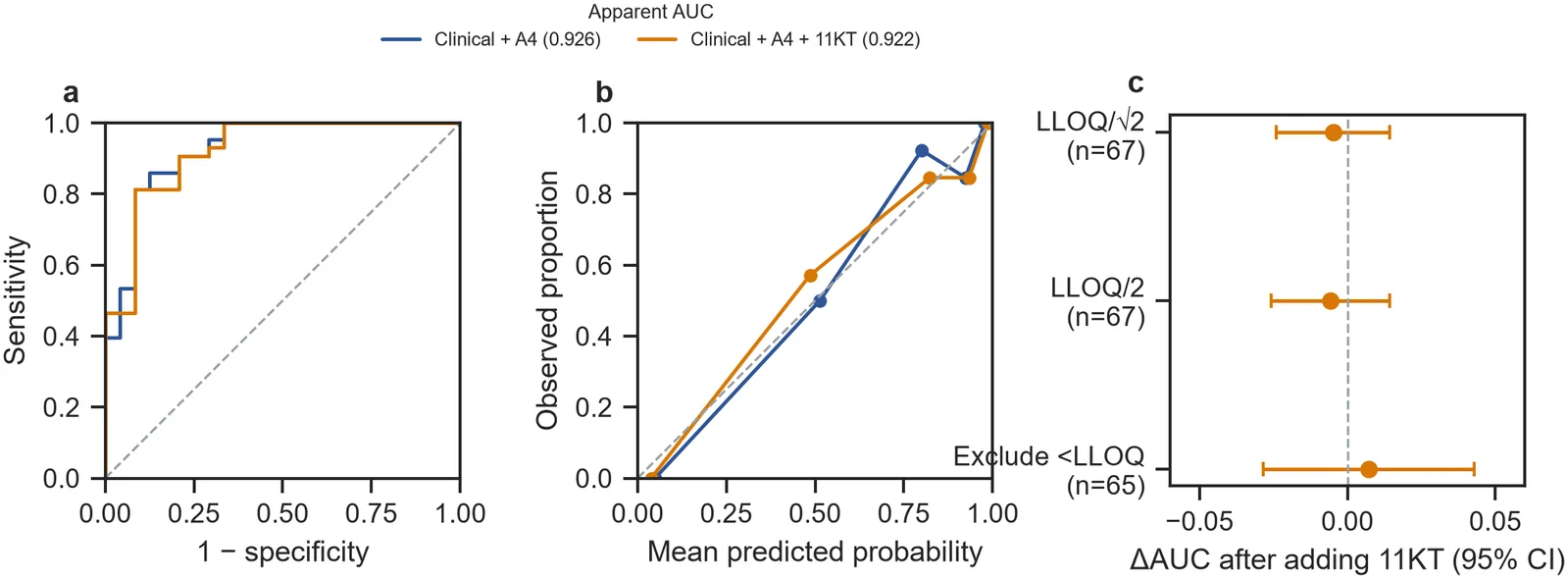
Primary incremental-value analysis. **(a)** Apparent ROC curves for the demographic/puberty + A4 ridge model with and without 11KT. **(b)** Apparent calibration across fifths of predicted probability. **(c)** ΔAUC after adding 11KT under primary and LLOQ sensitivity analyses; all confidence intervals are paired DeLong intervals. The central conclusion is that 11KT did not provide stable incremental discrimination beyond A4.

The single-marker AUC for 11KT was 0.875 (95% CI, 0.787–0.962), whereas the AUC for A4 was 0.906 (**Table 6A**; **Figure 4**). In exploratory steroid-only models, adding 11KT to 17-OHP modestly improved AUC (0.815 to 0.871) but without significant DeLong improvement. When A4 was included, 11KT provided minimal incremental value, with no improvement in discrimination, model fit, or calibration, suggesting substantial overlap between 11KT and A4 (**Table 6B**; **Figure 5**). In the primary ridge analysis, adding log2(11KT) to the demographic/puberty + A4 model changed the apparent AUC from 0.926 to 0.922 (ΔAUC = −0.005; 95% CI, −0.024 to 0.014; P = 0.620). The bootstrap-corrected AUC changed from 0.912 to 0.903, and the corrected calibration slope decreased from 0.835 to 0.695 (**Table 6C**; **Figure 6**).

**Table 6A:** Exploratory single-marker AUC comparisons for overall disease-control status in 21-OHD.

Biomarker	AUC (95% CI)	ΔAUC vs. 11KT (95% CI)	DeLong P
11KT	0.875 (0.788–0.962)	—	—
17-OHP	0.815 (0.706–0.924)	−0.060 (−0.152–0.032)	0.202
A4	0.906 (0.834–0.978)	0.031 (−0.047–0.109)	0.434
T	0.886 (0.810–0.961)	0.011 (−0.072–0.094)	0.802

**Table 6B:** Secondary exploratory steroid-only comparisons (not used as the primary multivariable inference).

Comparison	AUC ref./ext.	ΔAUC (95% CI)	DeLong P	LRT P	Brier ref./ext.	Corrected AUC ref./ext.	Corrected slope ref./ext.
17-OHP → + 11KT	0.815/0.871	0.056 (−0.018–0.130)	0.137	0.001	0.169/0.132	0.816/0.865	1.016/0.940
17-OHP + A4 → + 11KT	0.908/0.915	0.007 (−0.010–0.024)	0.439	0.359	0.104/0.104	0.901/0.898	0.916/0.774
17-OHP + T → + 11KT	0.894/0.908	0.014 (−0.014–0.041)	0.334	0.056	0.129/0.114	0.886/0.893	0.922/0.823
17-OHP + A4 + T → + 11KT	0.925/0.929	0.004 (−0.006–0.014)	0.449	0.452	0.101/0.100	0.914/0.908	0.859/0.670

**Table 6C:** Primary demographic/puberty + A4 ridge model with versus without 11KT.

Comparison	AUC ref./ext.	ΔAUC (95% CI)	DeLong P	Brier ref./ext.	Corrected AUC ref./ext.	Corrected Brier ref./ext.	Corrected calibration intercept/slope
Demographic/puberty + log2(A4) → + log2(11KT)	0.926/0.922	−0.005 (−0.024–0.014)	0.620	0.099/0.094	0.912/0.903	0.106/0.103	0.104/0.217; 0.835/0.695

LLOQ sensitivity analyses yielded the same qualitative conclusion (**Table 6D**). The ridge coefficients and bootstrap percentile 95% confidence intervals for the primary models are shown in **Table 6E**. With LLOQ/2 substitution, adding 11KT changed the AUC from 0.926 to 0.921 and the corrected slope from 0.835 to 0.689. After excluding two below-LLOQ observations, the AUC changed from 0.920 to 0.928, but the extended-model corrected calibration slope was 0.256. No handling rule produced a significant ΔAUC or stable calibration improvement. The 0.662 ng/mL single-marker cutoff remains exploratory and is not a clinical decision threshold.

**Table 6D:** LLOQ sensitivity analyses for the primary demographic/puberty + A4 ridge model.

11KT handling	n/events	AUC ref./ext.	ΔAUC (95% CI)	DeLong P	Corrected AUC ref./ext.	Corrected Brier ref./ext.	Corrected intercept/slope ref./ext.
Primary: LLOQ/√2	67/43	0.926/0.922	−0.005 (−0.024–0.014)	0.620	0.912/0.903	0.106/0.103	0.104/0.217; 0.835/0.695
Sensitivity: LLOQ/2	67/43	0.926/0.921	−0.006 (−0.026–0.014)	0.569	0.912/0.901	0.106/0.103	0.104/0.222; 0.835/0.689
Sensitivity: exclude < LLOQ	65/42	0.920/0.928	0.007 (−0.028–0.043)	0.691	0.906/0.908	0.108/0.100	0.097/0.334; 0.890/0.256

**Table 6E:** Ridge coefficients and bootstrap percentile 95% CIs for the primary demographic/puberty + A4 models.

Term	Demographic/puberty + log2(A4): β; OR (95% CI)	Demographic/puberty + log2(A4) + log2(11KT): β; OR (95% CI)
Intercept	0.521; 1.685 (0.149–12.008)	−0.168; 0.846 (0.100–5.760)
Age (per year)	0.145; 1.156 (0.904–1.658)	0.242; 1.274 (0.997–1.786)
Male sex	0.352; 1.422 (0.559–3.092)	0.275; 1.317 (0.541–2.976)
Tanner ≥2	−0.250; 0.779 (0.344–1.842)	−0.266; 0.767 (0.350–1.867)
log2(A4)	0.968; 2.633 (2.075–4.334)	0.626; 1.871 (1.273–3.842)
log2(11KT)	Not included	0.540; 1.716 (0.926–4.679)

Values are median [interquartile range (IQR)] or n (%). SW, classic salt-wasting; SV, classic simple-virilizing. AUC, area under the receiver operating characteristic curve; CI, confidence interval; GMR, geometric mean ratio; LRT, likelihood ratio test; OR, odds ratio; ext., extended model; ref., reference model.

### 3.6 Sensitivity analyses accounting for sex and pubertal stage

**Figure 7:**
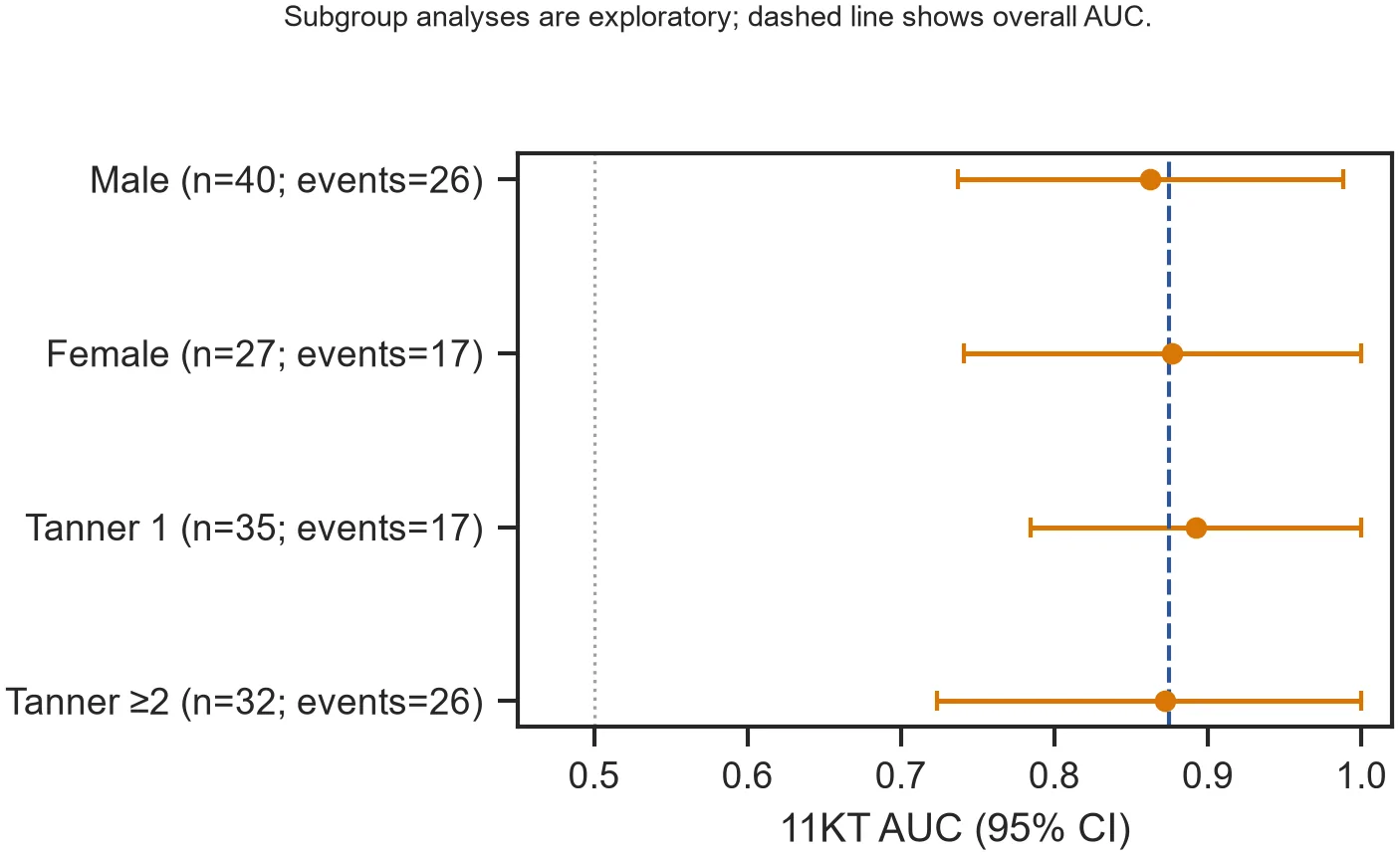
Exploratory subgroup AUCs for 11KT by sex and Tanner group. Points show AUCs with 95% CIs; the dashed line shows the overall 11KT AUC. Confidence intervals are wide because of the limited stratum-specific sample sizes.

Sex distribution was similar between well-controlled and suboptimally controlled children (male participants, 58.3% versus 60.5%; P = 1.000), whereas suboptimally controlled children were older and had a higher Tanner stage (P < 0.001 and P = 0.003, respectively; **Table 4**). Exploratory 11KT AUCs were 0.863 in male participants, 0.876 in female participants, 0.892 in Tanner stage 1, and 0.872 in Tanner stage ≥2 (**Figure 7**). The corresponding confidence intervals were wide, precluding precise subgroup comparisons.

## 4 Discussion

In this pediatric cohort, a single standardized morning 11KT concentration was higher in children with 21-OHD than in healthy controls and was strongly associated with retrospectively adjudicated overall disease-control status. The clinical interpretation is nevertheless constrained by the comparative analyses. A4 had a numerically higher single-marker AUC, and adding 11KT to age, sex, Tanner group, and A4 did not improve internally validated discrimination. The lower corrected calibration slope after adding 11KT, together with concordant LLOQ sensitivity analyses, argues against a stable incremental contribution in this cohort.

The association is biologically coherent with the adrenal origin and androgenic activity of 11-oxygenated C19 steroids. These steroids constitute a major circulating androgen pool in classic 21-OHD, are elevated in treated children with androgen excess, and have been associated with adrenal volume and testicular adrenal rest tumors (6–8). Our case-control and overall disease-control contrasts extend this evidence to a Chinese pediatric cohort. They support the biological relevance of 11KT, but they do not by themselves show that 11KT tracks treatment response over time.

The strong adjusted correlation between 11KT and A4 provides a parsimonious explanation for the limited incremental performance. Similar overlap has been reported in adults with classic CAH, in whom close correlations among adrenal steroids left the superiority of 11-oxygenated androgens over conventional markers unresolved (15). By contrast, Jha et al. showed that 11-oxygenated androgens were informative when 17-OHP and A4 were discordant (4). These observations are compatible rather than contradictory: 11KT may be useful in selected discordant cases, whereas our whole-cohort incremental analysis showed no general improvement beyond A4.

Disease control may also reflect shifts across several androgen pathways rather than the concentration of one analyte. A recent steroid-profiling study identified puberty as a risk factor for poor control and reported relative classical-pathway dominance among poorly controlled pubertal patients (16). Together, however, the findings suggest that pubertal state, pathway balance, and endpoint definition can modify biomarker interpretation. Our similar subgroup AUC point estimates should therefore be viewed as exploratory because their confidence intervals were wide.

Timing and developmental stage are additional sources of variation. The 11-oxygenated androgens show circadian and treatment-related fluctuations, and pediatric reference intervals vary with age and pubertal development (10, 12, 17). Salivary 11KT correlates closely with plasma 11KT and offers a potential route to repeated sampling (18). Standardized 08:00 premedication collection is therefore a strength of our study, but one measurement cannot summarize hormone exposure across a 12-month clinical observation period.

These considerations also limit threshold-based interpretation. Reviews of CAH monitoring emphasize that biochemical targets are not standardized and that growth, skeletal maturation, pubertal development, medication timing, and clinical safety remain essential to assessment (9, 12, 19). Recent modeling indicates that 17-OHP and A4 often provide similar information when sampling context and treatment regimen are considered (11). The 0.662 ng/mL 11KT cutoff derived in our sample was evaluated in the same small cohort and must not be interpreted as a clinical decision threshold. Prospective studies with repeated sampling, predefined outcomes, and external validation are needed before an age- and puberty-aware 11KT rule can be proposed.

Analytical constraints further narrow the inferences that can be drawn from the extended steroid panel. Of the 133 available 11KDHT measurements, 92 were reported at exactly 0.050 ng/mL. We therefore retained these analytes only for descriptive transparency and excluded them from quantitative inference. This decision prevents the reporting-floor pattern from being misinterpreted as biological clustering.

Strengths of this study include standardized premedication sampling, endpoint adjudication masked to steroid results, high initial adjudicator agreement, a prespecified incremental comparison, complete bootstrap refitting, and explicit LLOQ sensitivity analyses. Important limitations remain. However, the retrospective 12-month adjudication period may introduce temporal discordance with the single 11KT measurement obtained at the index visit. The single index measurement was compared cross-sectionally with a retrospective composite endpoint spanning 12 months, creating temporal mismatch and precluding longitudinal inference. The composite endpoint is not an independent biological gold standard, the single-center sample was small, and internal validation cannot replace external validation.

In conclusion, serum 11KT is associated with the cross-sectional overall disease-control phenotype in children with classic 21-OHD, but its information substantially overlaps that of A4. The present data support 11KT as a contextual biomarker for further study, particularly when conventional markers and clinical findings disagree. They do not support replacing A4, using the in-sample cutoff for clinical decisions, or inferring longitudinal treatment response from a single measurement.

## Data Availability

The original contributions presented in the study are included in the article/**Supplementary Material**. Further inquiries can be directed to the corresponding authors.
