# Supplementary material for: Serum 11-ketotestosterone is associated with overall disease-control status but adds limited information beyond androstenedione in children with 21-hydroxylase deficiency
Source: Front Endocrinol (Lausanne). 2026 Sep 8;17:1913565. doi: 10.3389/fendo.2026.1913565 (PMC13593492; doi:10.3389/fendo.2026.1913565)
Supplement: Supplementary file 1 [file Table1.docx]

# Supplementary Material

## Supplementary analytical validation and lower-limit audit

Supplementary Tables S1–S2 distinguish endpoint-component overlap, and initial adjudicator agreement.

**Supplementary Table S1. Mutually exclusive overlap patterns of unmet endpoint components among 43 suboptimally controlled children**

| **Exact component pattern** | **n** | **% of 43** |
| --- | --- | --- |
| 1 only | 2 | 4.7 |
| 2 only | 2 | 4.7 |
| 3 only | 7 | 16.3 |
| 4 only | 2 | 4.7 |
| 5 only | 5 | 11.6 |
| 1 + 2 | 1 | 2.3 |
| 1 + 5 | 1 | 2.3 |
| 2 + 3 | 2 | 4.7 |
| 3 + 5 | 5 | 11.6 |
| 4 + 5 | 5 | 11.6 |
| 1 + 2 + 5 | 3 | 7.0 |
| 1 + 4 + 5 | 2 | 4.7 |
| 2 + 3 + 5 | 2 | 4.7 |
| 3 + 4 + 5 | 4 | 9.3 |

Component codes: 1, uncontrolled virilization; 2, clinical glucocorticoid excess; 3, adrenal crisis/central precocious puberty/testicular adrenal rest tumor; 4, abnormal linear growth; 5, abnormal bone-age progression. Mutually exclusive pattern counts sum to all 43 suboptimally controlled children.

**Supplementary Table S2. Initial blinded adjudicator agreement for overall disease-control status**

| **Adjudicator 1** | **Adjudicator 2: suboptimal** | **Adjudicator 2: well controlled** | **Total** |
| --- | --- | --- | --- |
| Suboptimal | 41 | 2 | 43 |
| Well controlled | 1 | 23 | 24 |
| Total | 42 | 25 | 67 |

Observed agreement was 64/67 (95.5%); Cohen's κ=0.904. All three discordant cases underwent independent third-expert adjudication.
